# A Semi-supervised Learning-Based Diagnostic Classification Method Using Artificial Neural Networks

**DOI:** 10.3389/fpsyg.2020.618336

**Published:** 2021-01-20

**Authors:** Kang Xue, Laine P. Bradshaw

**Affiliations:** ^1^NWEA, Portland, OR, United States; ^2^Department of Educational Psychology, University of Georgia, Athens, GA, United States

**Keywords:** cognitive diagnostic classification, artificial neural networks, semi-supervised learning, machine learning, co-training algorithm

## Abstract

The purpose of cognitive diagnostic modeling (CDM) is to classify students' latent attribute profiles using their responses to the diagnostic assessment. In recent years, each diagnostic classification model (DCM) makes different assumptions about the relationship between a student's response pattern and attribute profile. The previous research studies showed that the inappropriate DCMs and inaccurate Q-matrix impact diagnostic classification accuracy. Artificial Neural Networks (ANNs) have been proposed as a promising approach to convert a pattern of item responses into a diagnostic classification in some research studies. However, the ANNs methods produced very unstable and unappreciated estimation unless a great deal of care was taken. In this research, we combined ANNs with two typical DCMs, the deterministic-input, noisy, “and” gate (DINA) model and the deterministic-inputs, noisy, “or” gate (DINO) model, within a semi-supervised learning framework to achieve a robust and accurate classification. In both simulated study and real data study, the experimental results showed that the proposed method could achieve appreciated performance across different test conditions, especially when the diagnostic quality of assessment was not high and the Q-matrix contained misspecified elements. This research study is the first time of applying the thinking of semi-supervised learning into CDM. Also, we used the validating test to choose the appropriate parameters for the ANNs instead of using typical statistical criteria.

## 1. Introduction

The purpose of cognitive diagnostic modeling (CDM; Templin and Henson, 2006) or diagnostic measurement is to provide students' skill/knowledge/attributes mastery status (mastery or non-mastery) through their responses to items from carefully designed assessments. Because of the ability to provide educators diagnostic feedback from students' assessment results, CDM has been the focus of much research in the last decade. Various types of diagnostic classification models (DCMs), such as the deterministic inputs, noisy and gate (DINA; Junker and Sijtsma, 2001), the reparametrized unified model/fusion model (RUM; Hartz, 2002), and the log-linear cognitive diagnosis model (LCDM; Henson et al., 2009), are designed based on different cognitive theories or assumptions about the relationship between a student's response pattern and attribute profile.

A principal research question of the previous research studies in CDM is which model better describes the data. When analysing a particular assessment dataset, selecting inappropriate DCMs (model misspecification) impacts the classification accuracy and parameter estimation. For example, when the attributes measured by an assessment are non-compensatory, which indicates that non-mastery on one attribute cannot be compensated by mastery on another attribute, selecting a compensatory model will decrease the performance of classification and measurement. DINA model and DINO (Templin and Henson, 2006) model achieved worse fit than did the other more relaxed DCMs, such as G-DINA (DeCarlo, 2011), LCDM, and RUM because both DINA and DINO might be too restrictive to reflect actual students' knowledge status (Yamaguchi and Okada, 2018). Some recent research studies (Chiu and Köhn, 2019; Yamaguchi and Okada, 2020; Zhan, 2020) started to apply the non-compensatory or conjunctive DCM, DINA model, and the compensatory or disjunctive DCM, DINO model, to build up a more general item response function (IRF) for CDM. However, these methods still require pre-data analysis procedure and assumptions of IRF to determine the hyperparameters contained in the mixture (or hybrid) CDM.

A Q-matrix indicates the relationship between items and attributes in an assessment. Q-matrices are often carefully designed by assessment experts, whereas some existing research and their experimental results have shown that Q-matrices constructed by content experts do not always reflect the relationship precisely and may require empirically-driven modifications (Bradshaw et al., 2014; Tjoe and de la Torre, 2014). In CDM, the diagnostic quality of an item indicates the discriminating power of the item to determine the success of the diagnosis. The item with high discriminating refers to that students who have mastered the attributes required by the item are expected to have a high probability of responding to the item correctly, while students who have not are expected to have a low probability. Items with low discriminating power compromise the accuracy of the estimate of student attribute mastery. In the previous research studies, the performances of all DCMs are sensitive to either the diagnostic quality of items or the accuracy of Q-matrices (Kunina-Habenicht et al., 2012; Liu et al., 2017).

Because of the increase of data size and development of computational power, artificial neural networks (ANNs; Goodfellow et al., 2016) have been proposed as an attractive approach to convert a pattern of item responses into a diagnostic classification (Cui et al., 2016; Guo et al., 2017; Paulsen, 2019; Xue, 2019). An ANN is a computational system inspired by biological neural systems for information processing in animals' brains. An ANN is built on inputs being translated to outputs through a series of neuron layers. It consists of three types of layers: an input layer, hidden layer(s), and an output layer. Each layer consists of a number of neurons (or nodes), and each node is connected to the nodes in the next layer. Each layer (except for the input layer) uses the output of its previous layer as the input. Supervised learning ANNs were applied in some research studies (Cui et al., 2016; Guo et al., 2017; Paulsen, 2019). To train the supervised learning ANNs, the ideal response patterns were set as the input layer and the associated attribute profiles as the output layer. Cui et al. (2016) hypothesized DINA model with both slipping and guessing equalling to 0 to synthesize ideal responses to train a multilayer perceptron (MLP). The experimental results showed that the classification accuracy of the supervised learning ANNs was not appreciated even in the simulated study. Another disadvantage of applying supervised learning ANNs for CDM is how to create the ideal response patterns using a DCM because both DCM and parameters are difficult to hypothesize. In addition to supervised learning ANNs, Cui et al. (2016) used one type of unsupervised learning ANNs, self-organizing map (SOM), to classify test-takers into different latent groups for CDM. One disadvantage of the unsupervised learning ANNs is that some further data analysis approaches are required to label the clusters. For example, although cluster analysis can place test-takers into different latent groups, *post hoc* techniques are required to discern the attributes from these latent groups. To do cluster labeling, Xue (2018) proposed a modified autoencoder network with a sparsely connected decoder explained the code layer outputs by using a part of the Q-matrix information. However, in both research studies, the unsupervised learning ANNs cannot yield comparable classification results compared with the DCMs, especially when the diagnostic quality of the assessment was not high. In addition, the ANNs methods produced very unstable and unappreciated estimation unless a great deal of care was taken to conduct sensitivity analyses (Briggs and Circi, 2017).

Regarding the disadvantages in supervised leaning ANNs and unsupervised learning ANNs, in this research, semi-supervised learning thinking is introduced to provide reasonable labels for ANN training and provide accurate and robust classification under different test conditions. In the machine learning field, semi-supervised learning (Zhu, 2005) concerns the study of how computers and natural systems learn in the presence of both labeled and unlabeled data, and it is somewhere between supervised learning and unsupervised learning. The research goal of semi-supervised learning is to understand how combining labeled and unlabeled data change the learning behavior, and design algorithms that take advantage of such a combination. Semi-supervised learning is a great interest in a wide range of applications, such as image search (Fergus et al., 2009), natural language parsing (Liang, 2005), and speech analysis (Liu and Kirchhoff, 2014) because the labeled data is scarce or expensive.

In this research, we firstly applied the semi-supervised learning thinking into the ANNs-based CDM. Unlike the hybrid CDM research studies, which used DINA and DINO models in a mixture CDM, in this research, DINA and DINO models were contained in a semi-supervised learning framework to improve the accuracy and consistency of the ANN's classification. In the following sections, we will first briefly introduce the Co-Training method, which is the semi-supervised learning method we used in this framework. Then, we will describe the structure of the ANNs will. Additionally, we will illustrate the experimental results under simulated experiments to compare the proposed method and five different DCMs. Lastly, we will outline the benefits and challenges of this methodology are summarized and future research.

## 2. Method

### 2.1. Co-training Methods of Using DINA Model and DINO Model

As one typical semi-supervised learning method, Co-Training (Nigam and Ghani, 2000) methods use a pair of classifiers with separate views of the data to iteratively learn and generate additional training labels. Like the self-training scheme, Co-Training is a wrapper method and widely applicable to many tasks. Co-Training bears a strong resemblance to the self-training scheme because each classifier uses its most confident predictions on unlabeled instances to teach itself. Two classifiers operate on different views of one observation, and the success of CoTraining depends on the following two assumptions (Zhu and Goldberg, 2009): (1) each view alone is sufficient to make good classifications, given enough labeled data; (2) the two views are conditionally independent given the class label.

Inspired by the typical Co-Training method, in this research, we chose the DINA model and DINO model as two classifiers to operate on different views of one response pattern to an item. The DINA model is a non-compensatory, or conjunctive DCM means that a lack of one attribute cannot be compensated by the mastery of another attribute measured by an item. For each item, the DINA model classifies students into two groups: those who have mastered all the attributes required by the item and those who have not. The *j*th item response probability of the *i*th student can be written as:

(1) $$\begin{array}{l} P\left(y_{ij}=1|\xi_{ij},s_{j},g_{j}\right)={\left(1-s_{j}\right)}^{\xi_{ij}}g_{j}^{1-\xi_{ij}} \end{array}$$

where ξ_*ij*_ = 1 indicates the *i*th student has mastered all required attributes of *j*th item, and ξ_*ij*_ = 0 refers to non-mastery status; *s*_*j*_ and *g*_*j*_ are the slipping parameter and guessing parameter of the *j*th item.

In contrast to the DINA model, the DINO model is a compensatory or disjunctive DCM, which means that a non-mastery on one latent attribute can be compensated for by a mastery status on another attribute. The *j*th item response probability of the *i*th student can be written as:

(2) $$\begin{array}{l} P\left(y_{ij}=1|\omega_{ij},s_{j},g_{j}\right)={\left(1-s_{j}\right)}^{\omega_{ij}}g_{j}^{1-\omega_{ij}} \end{array}$$

where the latent response ω_*ij*_ = 1 indicates that the *i*th student has mastered at least one attribute measured by *j*th item, and ω_*ij*_ = 0 indicates the absence of all required attributes. Like DINA, *s*_*j*_ and *g*_*j*_ are the slipping parameter and guessing parameter of the *j*th item.

The reason for selecting the DINA model and the DINO model is to hold the two assumptions of successfully applying Co-Training. First, in an assessment, either the DINA model or the DINO model can be the correct model for different items. For example, both the DINA and DINO models are the correct models for a simple structure item, which only measures a single attribute. Thus, using either the DINA model or the DINO model is sufficient to make accurate classification results. Second, the DINA model and DINO model's item response functions are represented based on different assumptions on the relationship between response patterns and attribute profiles. When the true latent class labels of students are known, for one item, the students can be divided into two groups, DINA-type and DINO-type, respectively. Considering the local independence (Wang and Douglas, 2015), test-takers' item responses from these two groups are statistically independent conditional on the true latent class labels.

In this paper, given the response data and Q-matrix, the DINA model and the DINO model were fitted. For an individual test-taker, we use two labels *c*_*DINA*_ and *c*_*DINO*_. *c*_*DINA*_ was the estimated latent class under the assumption of using the DINA model, and *c*_*DINO*_ was the estimated latent class under the assumption of using the DINO model. *c*_*DINA*_ and *c*_*DINO*_ could be either the same or different. In this research, the One-Hot encoding method (Harris and Harris, 2015) was applied to the integer encoding *c*_*DINA*_ and *c*_*DINO*_ to create two new One-Hot representation vectors $c_{DINA}=\left\{c_{DINA}^{k}\right\}$ and $c_{DINA}=\left\{c_{DINA}^{k^{\prime }}\right\}$. $c_{DINA}^{k}$ and $c_{DINA}^{k^{\prime }}\in \left\{0,1\right\}$, and $\underset{k}{\sum }c_{DINA}^{k}=\underset{k^{\prime }}{\sum }c_{DINA}^{k^{\prime }}=1$. In machine learning, a One-Hot is a group of bits among which the legal combinations of values are only those with a single 1 bit and all the others 0 bits. For example, if there are 4 latent classes, the integer encoding labels 1, 2, 3, and 4 are converted to One-Hot encoding [0001], [0010], [0100], and [1000], respectively.

### 2.2. Semi-supervised Learning ANN for Diagnostic Classification

**Figure 1:**
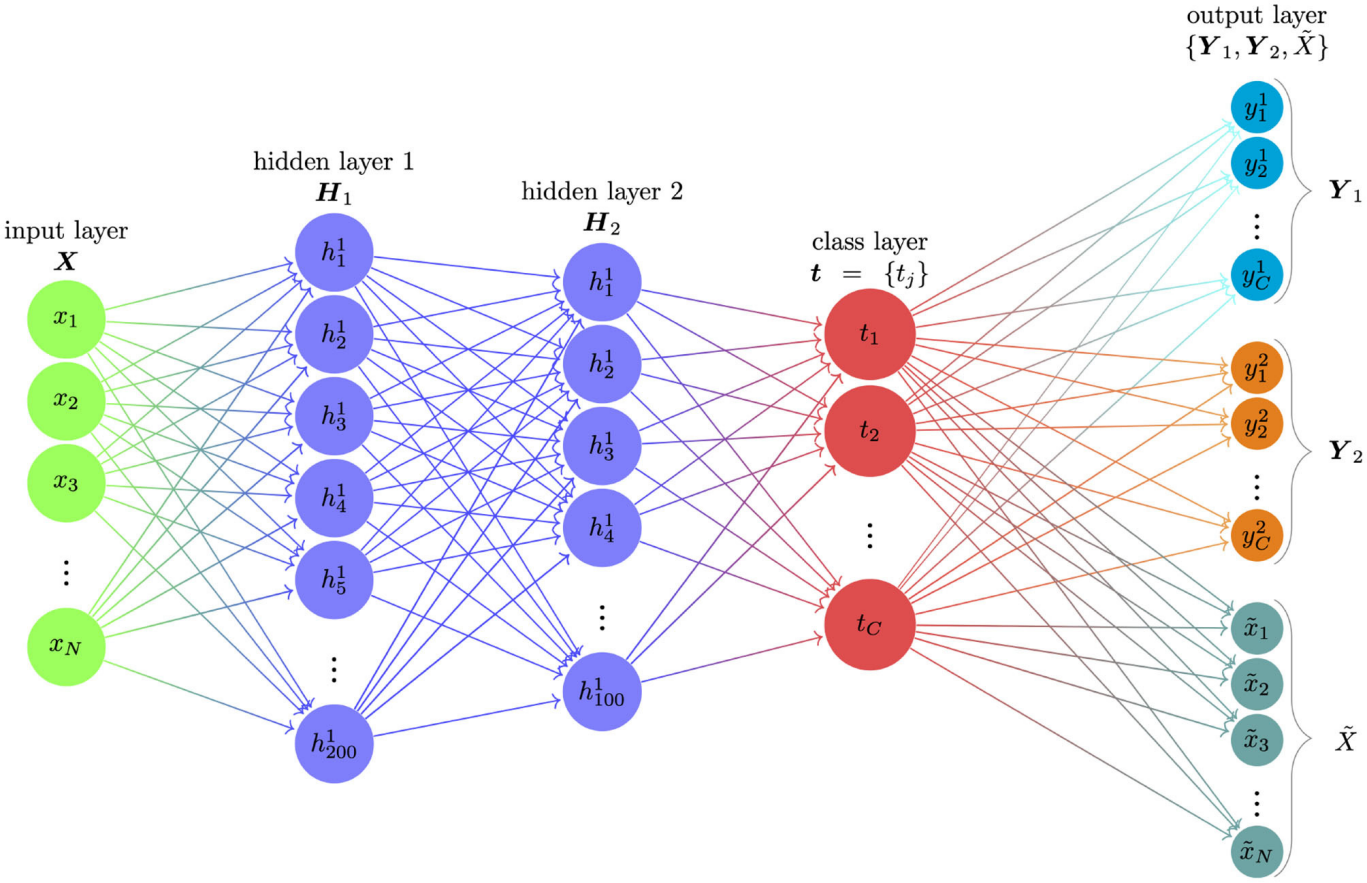
The structure of the proposed semi-supervised learning ANN. The proposed semi-supervised learning ANN consisted of one input layer, two hidden layers, one class layer and one output layer. The input ***X*** is response pattern of a test-taker; ***H***_1_ and ***H***_2_ are two hidden layers; ***t*** indicates the class layer; and the output layer consists of three parts: ***Y***_1_ is the classification under DINA assumption; ***Y***_2_ is the classification under DINO assumption; $\tilde{X}$ is the reconstructed response pattern of the test-taker.

As shown in Figure 1, the proposed semi-supervised learning ANN consisted of four parts: the input layer, two hidden layers, class layer, and the output layer. The number of nodes (the circles in Figure 1) on the input layer was equal to the number of items contained in the assessment. The number of nodes on the class layer was equal to the number of latent classes. To establish the relationship between the input and class nodes, we used two hidden layers (i.e., hidden layer 1 and hidden layer 2) to convert observed response patterns to latent classes. The numbers of nodes at these two hidden layers are 200 and 100. We use the Rectified linear unit (ReLU; Goodfellow et al., 2016) as the activation function for these two hidden layers and softmax function as the activation function for the class layer. In deep learning field, ReLU is widely used because the mathematical form of ReLU is very simple and efficient, and RelU can avoid a small derivative causing vanishing gradient problem. Softmax function is used for a multi-classification problem in ANNs. Since the number of nodes at the two hidden layers could be viewed as a hyperparameters of ANNs, we selected the two numbers (i.e., 200 and 100) for three reasons: (1) deep learning provides information-theoretically optimal approximation of a very wide range of functions and function classes used in mathematical signal processing (Grohs et al., 2019); (2) Lu et al. (2017) showed a universal approximation theorem for width-bounded ReLU networks: width-(*d* + 4) ReLU networks, where *d* is the input dimension, are universal approximators; (3) based on the validation test in our previous research studies using ANNs for psychometrics (Xue, 2018, 2019; Xue et al., 2020), these two values could achieve a balance between efficiency and accuracy.

In the supervised learning ANNs in CDM, only a single label was used for each observation. For example, when only using DINA classification as labels, the supervised learning ANN was used to train the standard softmax regression or a sigmoid regression (Pang et al., 2020) inputs to outputs without taking into account incorrect labels.The incorrect labels will impact the prediction performance of the ANNs for supervised learning ANNs. In contrast, the output layer in our proposed semi-supervised learning ANN consisted of three parts. The first part (output 1 or ***Y***_1_) corresponded to the DINA model classification, the second part (output 2 or ***Y***_2_) corresponded to the DINO model classification, and the third part was the reconstructed response pattern ($\tilde{X}$). The total number of output nodes was equal to two times of the number of hidden classes plus the number of items. For example, given an assessment with 30 items that measured a total of 4 attributes, the input layer ***X*** consisted of 30 input nodes (30 items), the class layer *t* consisted of 16 nodes (2^4^ = 16 latent classes), and the output layer $\left\{Y_{1},Y_{2},\tilde{X}\right\}$ consisted of 62 nodes (16 + 16 + 30).

Let ***X*** ∈ {0, 1}^***I***^ be the response patterns (*I* is the number of items), *c*_*DINA*_ and *c*_*DINO*_ be the One-Hot encoding of the DINA class labels and DINO class labels, respectively. Then we introduced into our ANN model the “true” latent class label (as opposed to the DINA and DINO class labels) as a latent multinomial variable *t* ∈ {0, 1}^*C*^, $\overset{C}{\underset{j}{\sum }}t_{j}=1$, where *C* is the number of latent classes. Like ***c***_*DINA*_ and ***c***_*DINO*_, *t* was also a One-Hot encoding label for each response pattern. The output of the class layers (or the input of the output layer) of our ANN was the posterior over ***t*** using the softmax regression. The *i*th element of ***t*** can be represented as:

(3) $$\begin{array}{l} P\left(t_{j}=1|X\right)=\frac{\tilde{P}\left(t_{j}=1|X\right)}{\sum_{j^{\prime }=1}^{C}\tilde{P}\left(t_{j^{\prime }}=1|X\right)}=\frac{\phi_{j}\left(X;\;w_{1}\right)}{\sum_{j^{\prime }=1}^{C}\phi_{j^{\prime }}\left(X;\;w_{1}\right)} \end{array}$$

where $\tilde{P}$ denotes the unnormalized probability distribution, Φ = {ϕ_*j*_(***X***; ***w***_1_)}, *j* ∈ {1, …, *C*} indicates the calculation from the input layer to class layer's output, and ϕ_*j*_(***X***; ***w***_1_) indicates the *j*th node's values on the class layer, the computation of ϕ_*j*_(***X***; ***w***_1_) is as follows:

(4) $$\begin{array}{l} \phi_{j}\left(X;\;w_{1}\right)=\sigma \left(\overset{100}{\underset{m=1}{\sum }}\overset{200}{\underset{n=1}{\sum }}w_{mj}^{H_{2}t}\;\max\left(w_{nm}^{H_{1}H_{2}}\;\max\left(w_{in}^{XH_{1}}X_{i},0\right),0\right)\right) \end{array}$$

where σ(·) is the softmax function, max(·, 0) is the ReLU function, $w_{1}=\left\{\left\{w_{in}^{XH_{1}}\right\},\left\{w_{nm}^{H_{1}H_{2}}\right\},\left\{w_{mj}^{H_{2}t}\right\}\right\}$ indicate the all the weights of the ANNs from the input layer to the class layer. $\left\{w_{in}^{XH_{1}}\right\}$ is the weights between input layer ***X*** and first hidden layer *H*_1_; $\left\{w_{nm}^{H_{1}H_{2}}\right\}$ is the weights between the first hidden layer *H*_1_ and second hidden layer *H*_2_; $\left\{w_{mj}^{H_{2}t}\right\}$ is the weights between the second hidden layer *H*_2_ and the class layer. ***w***_1_ needs to be estimated in the training of ANNs. Given the true label ***t***, the output 1 (DINA model classification) and output 2 (DINO model classification) can be modeled using another softmax with logits as follows:

(5) $$\begin{array}{l} \begin{array}{c} logit\left(P\left(Y_{1}|X\right)\right)=logit\left(P\left(c_{DINA}^{k}=1|X\right)\right)=\overset{C}{\underset{j=1}{\sum }}w_{jk}^{tY_{1}}t_{j}\\ logit\left(P\left(Y_{2}|X\right)\right)=logit\left(P\left(c_{DINO}^{k^{\prime }}=1|X\right)\right)=\overset{C}{\underset{j=1}{\sum }}w_{jk^{\prime }}^{tY_{2}}t_{j} \end{array} \end{array}$$

where the weights $w_{jk}^{tY_{1}}$ and $w_{jk^{\prime }}^{tY_{2}}$ learn the log-probability of the “true” label *j* as DINA class label *k* (the *k*th class in DINA classification) and as DINO class label *k*′ (the *k*′th class in DINA classification), respectively. Thus, in the proposed ANN, the joint relationship between input layer *x* and the *k*th node of ***Y***_1_ and *k*′th node of ***Y***_2_ can be represented as follows:

(6) $$\begin{array}{l} P\left(Y_{1},Y_{2}|X\right)=P\left(c_{DINA}^{k}=1,c_{DINO}^{k^{\prime }}=1|X\right)\\ \;=\overset{C}{\underset{j=1}{\sum }}P\left(c_{DINA}^{k}=1,c_{DINO}^{k^{\prime }}=1,t_{j}=1|X\right)\\ \;=\overset{C}{\underset{j=1}{\sum }}P\left(c_{DINA}^{k}=1|t_{j}=1\right)\\ \;P\left(c_{DINO}^{k^{\prime }}=1|t_{j}=1\right)P\left(t_{j}=1|X\right) \end{array}$$

where *P*(*t*_*j*_ = 1|***X***), $P\left(c_{DINO}^{k^{\prime }}=1|t_{j}=1\right)$, $P\left(c_{DINA}^{k}=1|t_{j}=1\right)$ are defined in Equations (3) and (5).

In addition to the difference between Co-Training labels and ***Y***_1_, ***Y***_2_, we also added a regularization term, $H\left(X,\tilde{X}\right)$, to encourage the classification to be perceptually consistent. ***X*** is the observed response pattern, and $\tilde{X}$ is the reconstructed response pattern corresponding to the estimated latent class. The $\tilde{X}$ can be calculated from the true label ***t*** as:

(7) $$\begin{array}{l} {\tilde{X}}_{i}=\overset{C}{\underset{j=1}{\sum }}w_{ji}^{t\tilde{X}}t_{j} \end{array}$$

where $w_{ij}^{t\tilde{X}}$ is the connection weights between *j*th class layer node and *i*th reconstructed output node. The weights between class layer and output layer of the ANNs is $w_{2}=\left\{\left\{w_{jk}\right\},\left\{w_{jk^{\prime }}\right\},\left\{w_{ji}\right\}\right\}$. We could perform training via stochastic gradient descent (SGD; Bottou and Bousquet, 2007) to minimize the following cost function:

(8) $$\begin{array}{l} \left\{w\right\}=\arg\min\left\{H\left(Y_{1},c_{DINA}\right)+H\left(Y_{2},c_{DINO}\right)+\lambda H\left(X,\tilde{X}\right)\right\} \end{array}$$

where {***w***} = {***w***_1_, ***w***_2_} indicates all the weights of the ANNs to be estimated, *H*(·, ·) is the cross-entropy to calculate the difference between ***Y***_1_ and One-Hot DINA labels ***c***_*DINA*_, and the difference between ***Y***_2_ and One-Hot DINO labels ***c***_*DINO*_, and the difference between observed response pattern ***X*** and reconstructed response pattern $\tilde{X}$. λ is a scaling parameter which was determined through a validation test (Xue et al., 2020).

Because of the large number of parameters contained in the deep learning structure, the random initialization of parameters may impact the optimization when the training sample size is not large enough. Thus, one concern of using ANNs for CDM is that using the feature extracted by deep learning through a single training is risky or sensitive to the starting points of the parameters (Briggs and Circi, 2017). Cui et al. (2016) only set a maximum number of iterations (e.g., 10,000) to stop training the supervised learning ANN in their research study. We applied two methods to deal with this issue. The first method was the early stopping, which is a simple, effective, and widely used approach to avoid overtraining the ANNs. The early stopping method is used to train on the training dataset but to stop training at the point when performance on a validation dataset starts to degrade. In addition, through the validating, we determined the scaling parameter in Equation (10). In our method, the whole data set was divided into two parts: the training dataset consisted of 80% observations, and the validating dataset consisted of the rest 20% observations. The second method was that we conducted 100 ANN trainings individually, produced a probability of latent class for each training, and then averaged the 100 probabilities as the final probability of the latent class for each test-taker.

## 3. Experimental Study

The aims of the experiment were (1) to examine the attribute profile estimation and classification accuracy of the proposed method under different test factors which are expected to affect the estimates' accuracy, and (2) to compare the proposed method with the performance of five DCMs: the DINA, DINO, G-DINA (De La Torre, 2011), LCDM (Henson et al., 2009), and RUM (Hartz, 2002). Thus, we conducted a simulation study under different assessment conditions with a variety of fixed factors and four manipulated factors.

### 3.1. Method

#### 3.1.1. Manipulated Factors

Using item by latent class matrix, we manipulated three assessment factors in the data generation for the simulation, including the number of items (20 or 30), number of attributes (three or four), and test diagnostic quality (high or mixed). When estimating the conditions, we also manipulated the Q-matrix accuracy (100 and 90% correct) as another factor expected to impact classification accuracy.

##### 3.1.1.1. Test Length and Number of Attributes

The number of items (20 or 30) and the number of attributes were selected to reflect the current real assessment applications, which often contained between 20 to 30 items and measured three or four attributes [e.g., MELAB data (Li and Suen, 2013); DTMR data (Bradshaw et al., 2014)]. For three attributes, we generated 20 items, and for four attributes, 20 and 30 items were generated, respectively. The three Q-matrices (i.e., 20 items measured 3 attributes, 20 items measured 4 attributes, and 30 items measured 4 attributes) for these conditions are shown in Supplementary Tables 1–3, respectively.

##### 3.1.1.2. Test Diagnostic Quality

Item discriminating power is another factor impact performance of DCMs shown in previous research studies (e.g., Cui et al. 2016; Roussos et al. 2005). The item discriminating power *d*_*i*_ is calculate as *d*_*i*_ = *p*(*x* = 1|α_1_) − *p*(*x* = 1|α_0_). α_0_ is the attribute pattern where none of the attributes measured by the *i*th item are mastered, and α_1_ is the attribute pattern where all attributes measured by the *i*th item are mastered. If *d*_*i*_ > 0.3, the Item *i* is a highly discriminating item, and if 0 < *d*_*i*_ ≤ 0.3, the Item *i* is a lowly discriminating item. In the assessments with high diagnostic quality, all items are of high discriminating power; in the assessments with mixed diagnostic quality, 50% items are of high discriminating power, and 50% items are of low discriminating power.

##### 3.1.1.3. Accuracy of Q-Matrix

Since the Q-matrices constructed by content experts do not always reflect the relationship precisely and may require empirically-driven modifications (Bradshaw et al., 2014; Tjoe and de la Torre, 2014), two levels of Q-matrix accuracy were also created for DCMs model fitting and Co-Training methods: 100% accuracy indicated that the Q-matrix were completely known; 90% accuracy indicated that 10% of elements in each Q-matrix were incorrect. We mis-specified the 10% elements in Q-matrix randomly drawing a Q-matrix entries and changing its value, with the constraint that each item must measure at least one attribute (i.e., a randomly drawn value of “1” for a simple structure item could not be changed to “0”). Such constrain makes there is no all zero q-vector (e.g., [0, 0, 0], [0, 0, 0, 0]) in Q-matrix.

#### 3.1.2. Generating Item Response Probabilities

Sample sizes of 1,000 were used for all conditions. The true class probabilities of correct response for the items in the item pools were simulated using the logic of a DCM with respect to the Q-matrix defining the item-class relationships and the probabilities following monotonicity constraints across non-equivalence classes on an item (i.e., masters of all attributes measured by the item having a higher probability of correct response than masters of a proper subset of these attributes; masters of no attributes measured by the item having a lower probability of correct response than masters of a proper subset of these attributes), but did not follow a particular existing DCM item response function (e.g., the LCDM or DINA function). Current DCM item response functions constrain the item response probabilities to be equal within all equivalence classes; our simulated data did not. Item-based equivalence classes are latent classes that have the same attribute profile, or the same pattern of mastery, for all attributes that are measured by the item. Conversely, item-based non-equivalence classes differ on the mastery status of one or more attributes measured by the item.

We simulated data using a general *I* × *C* item by latent class matrix (Xu and Zhang, 2016) according to DCM logic (i.e., defining latent classes by attribute profiles and specifying item-latent class relationships by the Q-matrix) without the specific mathematic representation of the item response function:

(9) $$\begin{array}{l} \Pi =\left[\begin{array}{cccc} \pi_{1,1} & \pi_{1,2} & \ldots & \pi_{1,C}\\ \pi_{2,1} & \pi_{2,2} & \ldots & \pi_{2,C}\\ \vdots & \vdots & \ddots & \vdots \\ \pi_{I,1} & \pi_{I,2} & \ldots & \pi_{I,C} \end{array}\right]. \end{array}$$

where the conditional probability that students in lth latent class answer *i*th item correctly *P*(*x*_*i*_ = 1|*c*) = π_*i,c*_, which is also known as item response probability (IRP) for each class. *I* indicated the number of items, *C* indicated the number of latent classes.

We denote π_*i*,α_0__, π_*i*,α_1__, and π_*i*,α_*p*__ as the IRPs for non-mastery group, mastery group, and partial mastery group, respectively. The mastery group contained students who mastered all of the attributes required by *i*th item, the partial mastery group contains students who only mastered a proper subset of attributes required by *i*th item, and the non-mastery group contained students who mastered none of the attributes required by *i*th item.

As shown in Table 1, when simulating response patterns to high discrimination items for the mastery group π_*i*,α_1__ were drawn from a uniform distribution *U*[0.65, 0.9]; for the non-mastery group π_*i*,α_0__ were drawn from a uniform distribution *U*[0.15, 0.35]; and for the partial mastery group π_*i*,α_*p*__ were drawn from a uniform distribution *U*[0.4, 0.6]. These draws yielded an average item discrimination value of 0.530 in 3 highly discriminating assessments. When simulating response patterns to low discrimination items, for the non-mastery group π_*i*,α_0__ were drawn from a uniform distribution *U*[0.2, 0.4]; for partial mastery group π_*i*,α_*p*__ were drawn from a uniform distribution *U*[π_*i*,α_0__, π_*i*,α_0__ + 0.2]; lastly for the mastery group (students who mastered all the attributes required by *i*th item) π_*i*,α_1__ were based on a uniform distribution *U*[π_*i*,α_*p*__, π_*i*,α_0__ + 0.3] for complex items and *U*[π_*i*,α_0__, π_*i*,α_0__ + 0.3] for simple items. This yielded an average item discrimination value of 0.387 in three mixed discriminating assessments.

**Table 1:** The table of selecting π_*i,c*_ for item by class matrix.

**Latent groups**	**High discrimination**	**Low discrimination**
Non-mastery π_*i,a*_0__	**U**[.15, .35]	**U**[.20, .40]
Partial-mastery π_*i*,α_*p*__	**U**[.40, .60]	**U**[π_*i*,*a*_0__, π_*i,a*_0__ + .15]
Mastery π_*i,a*_1__	**U**[.65, .90]	$\{\begin{array}{cc} U[\pi_{i,\alpha_{p}},\;\pi_{i,a_{0}}+0.30], & \text{Complex items}\\ U[\pi_{i,a_{0}},\;\pi_{i,a_{0}}+0.30], & \text{Simple items} \end{array}$

*For each item, π_i,a_0__, π_i,a_1__, and π_i,α_p__ indicate the π_i,c_ for non-mastery group, mastery group, and partial mastery group, respectively*.

By drawing true item parameters in this way, the π_*i,c*_s in our simulated data differs from IRPs simulated from the LCDM in that partial mastery classes with the same attribute pattern with respect to the measured attributes on a given item (the partial mastery item-based equivalence classes) have different true item response probabilities. The item response probabilities for these classes are, however, drawn from the same uniform distribution, so while they may be different values, they will be in the same range. Taking Item 10 that measures Attribute 1 and Attribute 2 as an example (as shown in Supplementary Table 4), Classes C2, C3, C6, and C7 are all partial mastery classes with respect to this item: Class C2 and C6 both have mastered Attribute 1 but not Attribute 2, and Class C3 and C7 has both mastered Attribute 2 and not Attribute 1. Under the LCDM, Class C2 and C6 would have the same IRP, while Class C3 and C7 would have the same IRP; under our generating model, the IRP for all four classes were drawn from the same interval, but the draws were different, resulting in, Class C2 having an IRP of 0.509, Class C3 having an IRP of 0.519, Class C6 having an IRP of 0.458, and Class C7 having an IRP of 0.429 (see Supplementary Table 4). For non-mastery equivalence classes and mastery equivalence classes, the true model did constrain draws to be equal within the interval (i.e., Class C1 and C5 have IRP values of 0.33 and Class C4 and C8 have IRP values of 0.891). Only for partial mastery item-based equivalence classes were they allowed to differ. The purpose of allowing this difference was to add some noise in the data while still controlling the item discrimination level (IRP of mastery group minus IRP of non-mastery group).

The values in the item by latent class matrix Π for the 6 item pools are shown in Supplementary Tables 4–9, respectively. These appendices showed that the DCMs primary monotonicity assumptions held. Namely, the mastery group has the greatest IRP, the non-mastery group has the lowest IRP, and the IRP of partial mastery groups lie between them. These appendices show this simulation procedure firstly held that 0.3 ≤ *d*_*i*_ < 0.75 for high discrimination items and 0.3 < *d*_*i*_ < 0.75 for low discrimination items; it also again shows the DCM monotonicity assumptions that the mastery group has a greater IRP than the non-mastery group held.

#### 3.1.3. Estimation

In our simulated study, as a comparison, five types of widely used DCMs were introduced as baselines to evaluate the diagnostic classification performance of the proposed framework. DINA and DINO models were selected as two baselines because they were the two classifiers used for Co-Training method. In addition, we chose three more general models, the G-DINA with identity link function (De La Torre, 2011), the LCDM with the logit link function (Henson et al., 2009), and the RUM (Hartz, 2002).

Results were analyzed in terms of classification accuracy of the five DCMs and proposed method under 12 different test conditions. Since in the proposed method, a validation test was introduced for early stop in the training procedure to avoid overtraining, the whole data set was divided to two parts: training dataset which contains 80% observations; and validating dataset which contains 20% observations. In the results shown in Tables 2–4, we list three types of the results of using the proposed ANN method:

1. ANN: the classification results of applying the trained ANN structure to the whole dataset containing training set and validation set;
2. ANN*: the classification results of applying the trained ANN structure to the training dataset;
3. ANN**: the classification results of applying the trained ANN structure to the validating dataset.

**Table 2:** Comparison of classification rates for three attributes using 20 items.

**Test condition**	**Methods**	**Quality accuracy**	**Q-matrix**	**Attribute 1**	**Attribute 2**	**Attribute 3**	**Class**
1	DINA	High	100%	0.949 (0.00)	0.864 (0.02)	0.957 (0.01)	0.778 (0.02)
	DINO			0.953 (0.01)	0.871 (0.02)	0.952 (0.02)	0.784 (0.04)
	LCDM			0.96 (0.00)	0.917 (0.00)	0.957 (0.00)	0.842 (0.01)
	G-DINA			0.96 (0.00)	0.917 (0.00)	0.957 (0.01)	0.842 (0.00)
	RUM			0.953 (0.01)	0.91 (0.00)	0.958 (0.00)	0.827 (0.00)
	ANN			0.956 (0.01)	0.915 (0.01)	0.957 (0.01)	0.834 (0.02)
	ANN*			0.962 (0.01)	0.921 (0.01)	0.964 (0.02)	0.851 (0.02)
	ANN**			0.945 (0.01)	0.901 (0.02)	0.942 (0.01)	0.818 (0.03)
2	DINA		90%	0.944 (0.00)	0.824 (0.01)	0.957 (0.00)	0.741 (0.02)
	DINO			0.946 (0.01)	0.852 (0.01)	0.944 (0.01)	0.757 (0.02)
	LCDM			0.956 (0.00)	0.897 (0.00)	0.958 (0.00)	0.819 (0.00)
	G-DINA			0.956 (0.00)	0.897 (0.00)	0.958 (0.01)	0.819 (0.00)
	RUM			0.949 (0.00)	0.879 (0.01)	0.958 (0.00)	0.794 (0.01)
	ANN			0.955 (0.01)	0.900 (0.02)	0.958 (0.02)	0.821 (0.02)
	ANN*			0.962 (0.01)	0.910 (0.02)	0.969 (0.02)	0.831 (0.03)
	ANN**			0.945 (0.01)	0.881 (0.02)	0.932 (0.04)	0.807 (0.04)
3	DINA	Mixed	100%	0.875 (0.00)	0.859 (0.01)	0.914 (0.00)	0.693 (0.01)
	DINO			0.863 (0.01)	0.864 (0.00)	0.896 (0.01)	0.665 (0.01)
	LCDM			0.879 (0.01)	0.884 (0.00)	0.913 (0.00)	0.712 (0.01)
	G-DINA			0.879 (0.00)	0.884 (0.00)	0.913 (0.00)	0.712 (0.00)
	RUM			0.873 (0.01)	0.9 (0.00)	0.917 (0.01)	0.724 (0.00)
	ANN			0.883 (0.01)	0.884 (0.02)	0.915 (0.01)	0.720 (0.01)
	ANN*			0.892 (0.01)	0.896 (0.01)	0.929 (0.02)	0.730 (0.02)
	ANN**			0.868 (0.01)	0.878 (0.02)	0.911 (0.01)	0.704 (0.02)
4	DINA		90%	0.878 (0.01)	0.85 (0.01)	0.906 (0.00)	0.676 (0.02)
	DINO			0.869 (0.00)	0.861 (0.00)	0.908 (0.00)	0.679 (0.01)
	LCDM			0.878 (0.00)	0.85 (0.00)	0.918 (0.00)	0.685 (0.01)
	G-DINA			0.877 (0.00)	0.85 (0.01)	0.918 (0.00)	0.684 (0.00)
	RUM			0.877 (0.00)	0.85 (0.01)	0.915 (0.00)	0.685 (0.01)
	ANN			0.874 (0.01)	0.888 (0.02)	0.908 (0.02)	0.704 (0.02)
	ANN*			0.889 (0.01)	0.901 (0.01)	0.923 (0.01)	0.719 (0.01)
	ANN**			0.867 (0.04)	0.871 (0.04)	0.890 (0.03)	0.683 (0.03)

ANN indicate the attribute profile estimation using the proposed method on whole data set. ANN* indicate the attribute profile estimation using the proposed method on the training data set. ANN** indicate the attribute profile estimation using the proposed method on the validation data set.

**Table 3:** Comparison of classification rates for four attributes using 20 items.

**Test condition**	**Methods**	**Quality accuracy**	**Q-matrix**	**Attribute 1**	**Attribute 2**	**Attribute 3**	**Attribute 4**	**Class**
5	DINA	High	100%	0.908 (0.02)	0.924 (0.03)	0.79 (0.02)	0.893 (0.02)	0.591 (0.03)
	DINO			0.909 (0.04)	0.928 (0.05)	0.858 (0.02)	0.899 (0.03)	0.653 (0.04)
	LCDM			0.918 (0.01)	0.929 (0.02)	0.858 (0.00)	0.919 (0.01)	0.67 (0.01)
	G-DINA			0.918 (0.01)	0.929 (0.01)	0.858 (0.01)	0.919 (0.00)	0.67 (0.01)
	RUM			0.923 (0.02)	0.921 (0.01)	0.853 (0.02)	0.917 (0.01)	0.664 (0.03)
	ANN			0.919 (0.01)	0.925 (0.01)	0.858 (0.03)	0.922 (0.04)	0.67 (0.03)
	ANN*			0.931 (0.02)	0.942 (0.01)	0.870 (0.03)	0.941 (0.01)	0.691 (0.03)
	ANN**			0.909 (0.03)	0.918 (0.02)	0.861 (0.01)	0.912 (0.04)	0.655 (0.03)
6	DINA		90%	0.909 (0.04)	0.922 (0.04)	0.74 (0.02)	0.886 (0.02)	0.56 (0.03)
	DINO			0.903 (0.04)	0.924 (0.02)	0.852 (0.03)	0.879 (0.04)	0.621 (0.04)
	LCDM			0.904 (0.01)	0.922 (0.00)	0.824 (0.01)	0.887 (0.01)	0.616 (0.01)
	G-DINA			0.904 (0.01)	0.922 (0.01)	0.824 (0.01)	0.887 (0.02)	0.616 (0.01)
	RUM			0.905 (0.02)	0.922 (0.02)	0.8 (0.02)	0.884 (0.01)	0.599 (0.03)
	ANN			0.912 (0.04)	0.923 (0.01)	0.862 (0.03)	0.89 (0.02)	0.648 (0.03)
	ANN*			0.924 (0.02)	0.931 (0.01)	0.877 (0.01)	0.901 (0.02)	0.657 (0.02)
	ANN**			0.903 (0.01)	0.917 (0.02)	0.853 (0.03)	0.883 (0.02)	0.632 (0.03)
7	DINA	Mixed	100%	0.854 (0.01)	0.836 (0.03)	0.824 (0.02)	0.851 (0.03)	0.503 (0.02)
	DINO			0.863 (0.02)	0.817 (0.04)	0.854 (0.02)	0.816 (0.04)	0.484 (0.04)
	LCDM			0.867 (0.01)	0.823 (0.01)	0.855 (0.02)	0.84 (0.03)	0.509 (0.01)
	G-DINA			0.867 (0.01)	0.824 (0.02)	0.855 (0.04)	0.84 (0.03)	0.51 (0.01)
	RUM			0.878 (0.03)	0.831 (0.02)	0.856 (0.03)	0.837 (0.04)	0.522 (0.03)
	ANN			0.864 (0.04)	0.842 (0.02)	0.857 (0.03)	0.859 (0.02)	0.531 (0.02)
	ANN*			0.879 (0.01)	0.855 (0.03)	0.870 (0.02)	0.871 (0.01)	0.550 (0.02)
	ANN**			0.853 (0.03)	0.839 (0.02)	0.826 (0.02)	0.850 (0.04)	0.504 (0.05)
8	DINA		90%	0.856 (0.04)	0.826 (0.02)	0.744 (0.01)	0.854 (0.01)	0.448 (0.02)
	DINO			0.854 (0.02)	0.817 (0.02)	0.855 (0.01)	0.851 (0.04)	0.503 (0.05)
	LCDM			0.865 (0.00)	0.817 (0.01)	0.776 (0.02)	0.844 (0.01)	0.469 (0.01)
	G-DINA			0.865 (0.02)	0.817 (0.01)	0.776 (0.00)	0.844 (0.01)	0.469 (0.01)
	RUM			0.864 (0.03)	0.821 (0.01)	0.855 (0.04)	0.84 (0.01)	0.509 (0.03)
	ANN			0.852 (0.02)	0.871 (0.02)	0.855 (0.03)	0.852 (0.01)	0.542 (0.04)
	ANN*			0.869 (0.03)	0.883 (0.02)	0.867 (0.01)	0.870 (0.00)	0.558 (0.03)
	ANN**			0.850 (0.04)	0.851 (0.02)	0.855 (0.02)	0.854 (0.03)	0.512 (0.05)

ANN indicate the attribute profile estimation using the proposed method on whole data set. ANN* indicate the attribute profile estimation using the proposed method on the training data set. ANN** indicate the attribute profile estimation using the proposed method on the validation data set.

**Table 4:** Comparison of classification rates for 4 attributes using 30 items.

**Test condition**	**Methods**	**Quality**	**Q-matrix accuracy**	**Attribute 1**	**Attribute 2**	**Attribute 3**	**Attribute 4**	**Class**
9	DINA	High	100%	0.937 (0.04)	0.938 (0.03)	0.814 (0.06)	0.892 (0.02)	0.641 (0.05)
	DINO			0.942 (0.01)	0.941 (0.08)	0.854 (0.11)	0.902 (0.08)	0.681 (0.12)
	LCDM			0.947 (0.01)	0.949 (0.00)	0.873 (0.02)	0.925 (0.02)	0.732 (0.02)
	G-DINA			0.947 (0.00)	0.949 (0.01)	0.873 (0.02)	0.925 (0.03)	0.732 (0.02)
	RUM			0.948 (0.02)	0.945 (0.00)	0.872 (0.04)	0.917 (0.03)	0.719 (0.05)
	ANN			0.949 (0.01)	0.944 (0.02)	0.872 (0.03)	0.916 (0.02)	0.722 (0.03)
	ANN*			0.955 (0.01)	0.952 (0.01)	0.880 (0.02)	0.935 (0.01)	0.741 (0.02)
	ANN**			0.942 (0.02)	0.940 (0.02)	0.860 (0.04)	0.903 (0.03)	0.711 (0.04)
10	DINA		90%	0.934 (0.03)	0.94 (0.02)	0.853 (0.04)	0.853 (0.03)	0.64 (0.04)
	DINO			0.935 (0.02)	0.924 (0.03)	0.855 (0.08)	0.874 (0.03)	0.644 (0.06)
	LCDM			0.948 (0.00)	0.946 (0.01)	0.858 (0.02)	0.92 (0.03)	0.708 (0.04)
	G-DINA			0.948 (0.02)	0.946 (0.01)	0.859 (0.03)	0.92 (0.03)	0.709 (0.03)
	RUM			0.945 (0.01)	0.945 (0.01)	0.869 (0.02)	0.915 (0.01)	0.713 (0.03)
	ANN			0.952 (0.02)	0.948 (0.01)	0.873 (0.02)	0.916 (0.01)	0.723 (0.02)
	ANN*			0.960 (0.02)	0.954 (0.02)	0.890 (0.01)	0.926 (0.01)	0.733 (0.02)
	ANN**			0.935 (0.03)	0.940 (0.04)	0.860 (0.02)	0.902 (0.04)	0.703 (0.04)
11	DINA	Mixed	100%	0.903 (0.03)	0.876 (0.03)	0.801 (0.01)	0.882 (0.02)	0.56 (0.02)
	DINO			0.911 (0.03)	0.884 (0.05)	0.858 (0.06)	0.858 (0.04)	0.586 (0.07)
	LCDM			0.912 (0.03)	0.886 (0.02)	0.857 (0.02)	0.88 (0.02)	0.616 (0.03)
	G-DINA			0.912 (0.02)	0.886 (0.01)	0.858 (0.02)	0.88 (0.01)	0.617 (0.02)
	RUM			0.9 (0.02)	0.884 (0.01)	0.858 (0.02)	0.871 (0.03)	0.592 (0.03)
	ANN			0.91 (0.01)	0.889 (0.02)	0.862 (.01)	0.881 (0.01)	0.616 (0.02)
	ANN*			0.916 (0.02)	0.898 (0.01)	0.869 (.02)	0.900 (0.01)	0.623 (0.02)
	ANN**			0.905 (0.02)	0.881 (0.03)	0.850 (.02)	0.881 (0.03)	0.605 (0.03)
12	DINA		90%	0.908 (0.03)	0.887 (0.03)	0.847 (0.01)	0.876 (0.03)	0.603 (0.02)
	DINO			0.906 (0.03)	0.883 (0.07)	0.852 (0.08)	0.836 (0.07)	0.566 (0.09)
	LCDM			0.908 (0.02)	0.891 (0.01)	0.863 (0.03)	0.868 (0.01)	0.605 (0.02)
	G-DINA			0.908 (0.01)	0.891 (0.02)	0.863 (0.03)	0.868 (0.01)	0.605 (0.02)
	RUM			0.905 (0.02)	0.891 (0.01)	0.864 (0.03)	0.861 (0.03)	0.602 (0.03)
	ANN			0.909 (0.01)	0.885 (0.02)	0.859 (0.01)	0.871 (0.02)	0.61 (0.02)
	ANN*			0.921 (0.01)	0.903 (0.02)	0.869 (0.01)	0.878 (0.01)	0.624 (0.01)
	ANN**			0.901 (0.03)	0.889 (0.02)	0.850 (0.01)	0.857 (0.03)	0.603 (0.03)

ANN indicate the attribute profile estimation using the proposed method on whole data set. ANN* indicate the attribute profile estimation using the proposed method on the training data set. ANN** indicate the attribute profile estimation using the proposed method on the validation data set.

The data simulation and five DCMs were conducted using the “CDM” package (George et al., 2016) in R. The proposed semi-supervised learning ANN was conducted using the “tensorflow” library (Pang et al., 2020) in Python. In the experimental study, we conducted 100 replications. In each replication, new response patterns were created based on the fixed values in the item by latent class matrices in Supplementary Tables 4–9.

### 3.2. Results

First, we tested the effects of the four assessment factors of test length, number of attributes, test diagnostic quality, and Q-matrix accuracy on the attribute profile and classification accuracy for the proposed method. Then we compared the proposed method to the five DCMs, under 12 different test conditions. Results are given in Tables 2–4.

#### 3.2.1. Classification Accuracy and Four Assessment Factors

We first focus on results for the proposed method. As mentioned in the Estimation session, ANN, ANN* and ANN** in Tables 2–4 indicate the classification accuracy on whole dataset (including training set and validating set), the training set and validating set, respectively. Results show that the proposed method (ANN) works reasonably well and has classification accuracy values >70% under 6 out of 12 assessment conditions (condition 1, 2, 3, 4, 9, and 10) when applying the trained ANN to the whole data set (i.e., ANN). Condition 1–4 are all four test conditions for the assessment measures 3 attributes using 20 items with either highly diagnostic quality or mixed diagnostic quality. Condition 9 and 10 are the two test conditions for assessment measures 4 attributes using 30 items with highly diagnostic quality. Results show classification accuracy increased in expected ways for the proposed method. Namely, average classification accuracy increases from 0.670 to 0.722 as test length increases from 20 to 30 for assessments measure 4 attributes (there is only one test length of assessment that measures 3 attributes); when the number of attribute measured decreases from 4 to 3 in assessment with 20 items, the average classification accuracy increases from 0.670 to 0.834; when the test diagnostic quality increases from mixed to high, the average classification accuracy increases from 0.621 to 0.736; and when the accuracy of Q-matrix increases from 90 to 100%, the average accuracy increases slightly from 0.675 to 0.682. In addition, we can see that ANN* always achieves the best performance with average classification accuracy 0.692, ANN** always achieves the worst performance with average classification accuracy 0.661, and ANN falls between ANN* and ANN** with average classification accuracy 0.678. The reason is that the parameters of ANN structure were trained based on the training dataset but not considered the validation dataset.

Next, we examine the results for the five DCMs. Results show that DINA model has classification accuracy values >70% under 2 out of 12 assessment conditions (condition 1 and 2); DINO model has classification accuracy values >70% under 2 out of 12 assessment conditions (condition 1 and 2); G-DINA has classification accuracy values >70% under 5 out of 12 test conditions (condition 1, 2, 3, 9, and 10); LCDM has classification accuracy values >70% under 5 out of 12 test conditions (condition 1, 2, 3, 9, and 10); and RUM has classification accuracy values >70% under 5 out of 12 test conditions (condition 1, 2, 3, 9, and 10). Condition 1 and 2 are two tests (high and mixed diagnostic quality) with 20 items measures 3 attributes and the Q-matrix accuracy is 100%; condition 3 is a test with high diagnostic quality consists of 20 items to measure 3 attribute but the Q-matrix accuracy is 90%; condition 9 and 10 are two tests (high and mixed diagnostic quality) with 30 items measures 4 attributes and the Q-matrix accuracy is 100%. We could also notice that the G-DINA and LCDM achieved almost the same classification results because the only difference between G-DINA and LCDM in the CDM::gdina() is the link function. We chose “identity” function for G-DINA and “logit” function for LCDM. In addition, like the proposed method, results show classification accuracy increased in expected way for the 5 DCMs. Namely, accuracy increases as test length increases, as the number of attribute measured decreases, as the test diagnostic quality increases, and as the accuracy of Q-matrix increases.

#### 3.2.2. Comparison Classification With 5 DCMs

Simulation results indicated that when using the proposed ANN, the classification rates were higher than rates from the DINA and DINO models, the two initial classifiers used in Co-Training. Compared to DINA and DINO models, at the attribute level, the average improvements of classification using ANN was 0.0218 and 0.0140, and at the class level (i.e., attribute profiles level), the average improvements were 0.0589 and 0.0432. Compared to the general models LCDM and G-DINA, which often achieved the best performance in classification, the performance of ANN was also better than these two methods. The improvements at the attribute level were 0.0056 and 0.0055 compared with LCDM and G-DINA models, respectively. At the class level, the improvements were 0.0130 and 0.0132.

The simulated study also indicated that when the Q-matrix became less accurate, the classification accuracy for each method dropped at both attribute level and latent class level when holding other test assessment factors. When the Q-matrix accuracy decreased to 90% accurate, at the attribute level, the average reductions of classification accuracy were 0.0071, 0.0055, 0.0114, 0.0114, 0.0095, and 0.0038 corresponding to DINA, DINO, LCDM, G-DINA, RUM, and our ANN methods, respectively. At the attribute pattern level, the average accuracy reductions were 0.0163, 0.0138, 0.0298, 0.0302, 0.0243, and 0.0075 for DINA, DINO, LCDM, G-DINA, RUM and, our ANN methods, respectively. From this observation, we could find that firstly, the relaxed models (LCDM, G-DINA, and RUM) were more sensitive to the accuracy of Q-matrix; secondly, the proposed ANN was more robust to the noise within the Q-matrix compared to the five DCMs.

Besides, high item discriminating was a positive impact on the classification accuracy of all six methods. When the discrimination of items decreased (from high to mixed), the classification rate dropped 0.0301, 0.0383, 0.0458, 0.0458, 0.0392, and 0.0397 for DINA, DINO, LCDM, G-DINA, RUM, and our ANN at the attribute level. The reductions were 0.0780, 0.1095, 0.1318, 0.1318, 0.1137, and 0.1158 for DINA, DINO, LCDM, G-DINA, RUM, and our ANN at the latent class level. The reason that our ANN method dropped more than DINA, DINO, and RUM (only at the attribute level) was that when the items were high discriminating, the improvement of classification rate using our ANN was more significant than using mixed discriminating items. Even though the performance of our ANN at both the attribute level and the latent class level was the best among the six diagnostic classification methods.

## 4. Conclusion

The purpose of this research is to solve two problems that exist in current supervised learning ANN methods and unsupervised learning ANNs: the supervised learning method requires ideal response pattern to train the model; the classification accuracy of unsupervised learning methods was not as good as DCMs. We designed a novel semi-supervised learning ANN to do diagnostic classification and evaluated the performances of the proposed method through a simulation study. In the proposed framework, we combined ANN with a semi-supervised learning method, the Co-Training method. To hold the two assumptions of successfully applying Co-Training, we used two DCMs, DINA, and DINO models, as the two classifiers.

In the simulated study, we compared the proposed method with five widely used DCMs, DINA, DINO, LCDM, G-DINA, and RUM. By varying the four assessment factors (item discrimination, Q-matrix accuracy, number of attributes, and items) which impact the performance of DCMs, the comparison results indicated some advantages of the proposed method.

The first advantage is that the proposed ANN method achieved comparable performance compared with the five DCMs even under the ideal assessment condition (high diagnostic quality and 100% Q-matrix accuracy). It means that the proposed ANN method could be used for providing reasonable cognitive diagnostic classification result without an appropriate DCM for an assessment.

The second advantage is that proposed ANN was robust to the Q-matrix mis-specification because the classification rate dropped less than the other five DCMs when the Q-matrix accuracy decreased to 90% accuracy. This advantage make the proposed method can be used for real large scale assessment because the Q-matrix of a large number of items can hardly be guaranteed to be 100% accurate.

The last advantage is that although the classification rates of the proposed method dropped more than DINA and DINO when the item discriminating power reduced, the proposed method was still more robust to the item discriminating reduction than the general DCMs. In other words, the proposed method finds a trade-off between classification accuracy and robustness to the noise.

Generally, the proposed method could demonstrated the ability to provide a reasonably accurate classification results which can be used for either providing diagnostic classification. In addition, the classification can be used to determine the relationship between items and latent class. Then, the relationship can help researchers to choose the appropriate DCM to fit the data and estimate both personal variable and item variables.

## 5. Discussion

Although the study demonstrates promise for using the proposed semi-supervised learning artificial neural networks, there are still some limitations. One concern of this study is that the current analysis only focused on the classification rate but not consider the item parameters, which are very important to provide appropriate item matching students' ability in an computer adaptive test or online adaptive learning environment. Another concern of this study is that the missing response was not considered in the proposed ANN. In the simulation, we assumed that all test-takers responded all items, but in real assessment, the missingness is a very common issue in CDM. The last concern is that although we introduced the validating test for early stop to avoid over training, this research did not evaluate the prediction performance of the proposed method. The reason is that in current CDM area, the research studies focus on explaining data not doing prediction on a new dataset. With regard to these three concerns, there will be three future research topics.

The first future study is that the classification results could be used to determine the item parameters to evaluate item discriminating power among students' mastery level for specific attributes or determine the relationship between items and attributes to explore the attribute structures. An appropriate difficulty that matches a student's momentary attribute profile is expected to encourage the student to complete the item.

The second future research direction is to convert the dichotomous response patterns to polychotomous response patterns by considering missing values into the input response pattern. Then a multiclass classification algorithm is applied to classify the latent classes by considering the missing values even the missingness is related to the latent class (i.e., non-ignorable missingness).

The last future research is to evaluate the prediction performance of the artificial neural network based cognitive diagnostic classification method, and compare the performance with the DCMs in doing prediction on new dataset, although DCMs are proposed to interpret the current dataset (i.e., training data). With regard to the knowledge in educational data mining (EDM), the prediction will consist of two directions: (1) how is the model's performance on predicting new test-takers' latent variables; (2) how is the model's performance on estimating new item's characteristics. For different directions, the ANN based method will be built up using different architecture.

## Data Availability Statement

The raw data supporting the conclusions of this article will be made available by the authors, without undue reservation.

## Author's Note

We designed a novel semi-supervised learning ANN to do diagnostic classification and evaluated the proposed method's performances through a simulation study. This research study is the first time applying the thinking of semi-supervised learning into CDM using artificial neural networks. The results show that even without an appropriate theoretical DCM, the proposed method can demonstrate the ability to provide comparable classification results compared with the theoretical DCMs. It means that the proposed ANN method could provide reasonable cognitive diagnostic classification results without an appropriate TDCM for an assessment. Besides, compared with the theoretical DCMs, the proposed method can be used for real large scale assessment because it is more robust to noisy assessment factors (e.g., inaccurate Q-matrix, low discriminative items).

## Author Contributions

All authors listed have made a substantial, direct and intellectual contribution to the work, and approved it for publication.

## Conflict of Interest

The authors declare that the research was conducted in the absence of any commercial or financial relationships that could be construed as a potential conflict of interest.
