# Supplementary material for: A Semi-supervised Learning-Based Diagnostic Classification Method Using Artificial Neural Networks
Source: Front Psychol. 2021 Jan 20;11:618336. doi: 10.3389/fpsyg.2020.618336 (PMC7856146; doi:10.3389/fpsyg.2020.618336)
Supplement: Supplementary file 1 [file Data_Sheet_1.PDF]

frontiers

Supplementary Material

1 Q-MATRICES USED IN DATA SIMULATION

Table S1. Q-matrix for 3 Attributes, 20 Items Test.

Item	Attribute 1	Attribute 2	Attribute 3
1	1	0	0
2	0	1	0
3	0	0	1
4	1	0	0
5	0	1	0
6	0	0	1
7	0	1	1
8	1	1	0
9	0	0	1
10	1	1	0
11	1	1	1
12	1	0	0
13	1	1	0
14	1	0	1
15	1	1	0
16	0	0	1
17	0	1	0
18	1	0	0
19	1	0	1
20	1	0	1

Table S2. Q-matrix for 4 Attributes, 20 Items Test.

Item	Attribute	Attribute 2	Attribute 3	Attribute 4
1	1	0	0	0
2	0	1	0	0
3	0	0	1	0
4	0	0	0	1
5	1	0	0	0
6	0	1	0	0
7	0	0	1	0
8	0	0	0	1
9	0	1	1	1
10	1	1	0	1
11	0	0	1	0
12	1	1	0	0
13	1	1	1	1
14	1	0	0	1
15	1	1	0	1
16	1	0	1	0
17	1	1	0	0
18	0	0	1	1
19	0	0	0	1
20	0	1	0	1

Table S3. Q-matrix for 4 Attributes, 30 Items Test.

Item	Attribute	Attribute 2	Attribute 3	Attribute 4
1	1	0	0	0
2	0	1	0	0
3	0	0	1	0
4	0	0	0	1
5	1	0	0	0
6	0	1	0	0
7	0	0	1	0
8	0	0	0	1
9	0	1	1	1
10	1	1	0	1
11	0	0	1	0
12	1	1	0	0
13	1	1	1	1
14	1	0	0	1
15	1	1	0	1
16	1	0	1	0
17	1	1	0	0
18	0	0	1	1
19	0	0	0	1
20	0	1	0	1
21	1	0	0	0
22	1	0	1	1
23	1	0	1	1
24	0	1	0	1
25	0	0	1	0
26	1	1	1	1
27	0	0	0	1
28	0	1	1	1
29	0	1	0	1
30	1	0	0	1

2 ITEM BY LATENT CLASS MATRICES UNDER DIFFERENT ASSESSMENT CONDITIONS

Table S4. Π of 3 Attributes, 20 Items, High Discrimination Test.

Item	C1<br>000	C2<br>100	C3<br>010	C4<br>110	C5<br>001	C6<br>101	C7<br>011	C8<br>111
1	.308*	.722***	.308*	.722***	.308*	.722***	.308*	.722***
2	.327*	.327*	.752***	.752***	.327*	.327*	.752***	.752***
3	.159*	.159*	.159*	.159*	.885***	.885***	.885***	.885***
4	.328*	.782***	.328*	.782***	.328*	.782***	.328*	.782***
5	.241*	.241*	.788***	.788***	.241*	.241*	.788***	.788***
6	.241*	.241*	.241*	.241*	.889***	.889***	.889***	.889***
7	.158*	.158*	.536**	.515**	.421**	.58**	.712***	.712***
8	.349*	.466**	.591**	.81***	.349*	.578**	.539**	.81***
9	.292*	.292*	.292*	.292*	.814***	.814***	.814***	.814***
10	.33*	.509**	.519**	.891***	.33*	.458**	.429**	.891***
11	.196*	.538**	.559**	.405**	.496**	.552**	.443**	.73***
12	.233*	.686***	.233*	.686***	.233*	.686***	.233*	.686***
13	.243*	.483**	.474**	.708***	.243*	.43**	.428**	.708***
14	.174*	.453**	.174*	.572**	.409**	.85***	.488**	.85***
15	.225*	.512**	.441**	.874***	.225*	.426**	.551**	.874***
16	.169*	.169*	.169*	.169*	.816***	.816***	.816***	.816***
17	.205*	.205*	.746***	.746***	.205*	.205*	.746***	.746***
18	.24*	.854***	.24*	.854***	.24*	.854***	.24*	.854***
19	.276*	.562**	.276*	.562**	.559**	.839***	.488**	.839***
20	.273*	.542**	.273*	.4**	.495**	.745***	.444**	.745***

Note. * indicates the π_{ic} for non mastery group, ** indicates the π_{ic} for partial mastery group, *** indicates the π_{ic} for mastery group. The binary vector (e.g., 001) under class name (e.g., C5) indicates the attribute profile of the latent class.

Table S5. Π of 3 Attributes, 20 Items, Mixed Discrimination Test.

Item	C1 000	C2 100	C3 010	C4 110	C5 001	C6 101	C7 011	C8 111
1	.378*	.579***	.378*	.579***	.378*	.579***	.378*	.579***
2	.349*	.349*	.81***	.81***	.349*	.349*	.81***	.81***
3	.331*	.331*	.331*	.331*	.537***	.537***	.537***	.537***
4	.269*	.786***	.269*	.786***	.269*	.786***	.269*	.786***
5	.179*	.179*	.722***	.722***	.179*	.179*	.722***	.722***
6	.393*	.393*	.393*	.393*	.654***	.654***	.654***	.654***
7	.193*	.193*	.538**	.559**	.405**	.496**	.84***	.84***
8	.224*	.464**	.446**	.753***	.224*	.429**	.483**	.753***
9	.178*	.178*	.178*	.178*	.688***	.688***	.688***	.688***
10	.247*	.317**	.287**	.447***	.247*	.375**	.253**	.447***
11	.225*	.56**	.424**	.512**	.441**	.426**	.551**	.874***
12	.333*	.361***	.333*	.361***	.333*	.361***	.333*	.361***
13	.312*	.477**	.455**	.853***	.312*	.563**	.49**	.853***
14	.15*	.559**	.15*	.488**	.551**	.828***	.526**	.828***
15	.295*	.328**	.352**	.409***	.295*	.387**	.348**	.409***
16	.284*	.284*	.284*	.284*	.711***	.711***	.711***	.711***
17	.284*	.284*	.512***	.512***	.284*	.284*	.512***	.512***
18	.221*	.347***	.221*	.347***	.221*	.347***	.221*	.347***
19	.397*	.531**	.397*	.53**	.423**	.633***	.417**	.633***
20	.169*	.469**	.169*	.531**	.464**	.846***	.438**	.846***

Note. * indicates the π_{ic} for non mastery group, ** indicates the π_{ic} for partial mastery group, *** indicates the π_{ic} for mastery group. The binary vector (e.g., 001) under class name (e.g., C5) indicates the attribute profile of the latent class.

Table S6. Π of 4 Attributes, 20 Items, High Discrimination Test.

Item	C1 0000	C2 1000	C3 0100	C4 1100	C5 0010	C6 1010	C7 0110	C8 1110	C9 0001	C10 1001	C11 0101	C12 1101	C13 0011	C14 1011	C15 0111	C16 1111
1	.308*	.722***	.308*	.722***	.308*	.722***	.308*	.722***	.308*	.722***	.308*	.722***	.308*	.722***	.308*	.722***
2	.327*	.327*	.752***	.752***	.327*	.327*	.752***	.752***	.327*	.327*	.752***	.752***	.327*	.327*	.752***	.752***
3	.159*	.159*	.159*	.159*	.885***	.885***	.885***	.885***	.159*	.159*	.159*	.159*	.885***	.885***	.885***	.885***
4	.328*	.328*	.328*	.328*	.328*	.328*	.328*	.328*	.782***	.782***	.782***	.782***	.782***	.782***	.782***	.782***
5	.241*	.788***	.241*	.788***	.241*	.788***	.241*	.788***	.241*	.788***	.241*	.788***	.241*	.788***	.241*	.788***
6	.241*	.241*	.889***	.889***	.241*	.241*	.889***	.889***	.241*	.241*	.889***	.889***	.241*	.241*	.889***	.889***
7	.265*	.265*	.265*	.265*	.819***	.819***	.819***	.819***	.265*	.265*	.265*	.265*	.819***	.819***	.819***	.819***
8	.33*	.33*	.33*	.33*	.33*	.33*	.33*	.33*	.676***	.676***	.676***	.676***	.676***	.676***	.676***	.676***
9	.179*	.179*	.449**	.408**	.466**	.591**	.578**	.539**	.528**	.599**	.531**	.542**	.509**	.519**	.722***	.722***
10	.224*	.593**	.58**	.538**	.224*	.559**	.405**	.496**	.552**	.443**	.464**	.753***	.446**	.429**	.483**	.753***
11	.178*	.178*	.178*	.178*	.688***	.688***	.688***	.688***	.178*	.178*	.178*	.178*	.688***	.688***	.688***	.688***
12	.191*	.447**	.493**	.79***	.191*	.453**	.572**	.79***	.191*	.409**	.488**	.79***	.191*	.56**	.424**	.79***
13	.276*	.426**	.551**	.579**	.475**	.533**	.419**	.477**	.455**	.563**	.49**	.562**	.562**	.559**	.488**	.839***
14	.284*	.542**	.284*	.4**	.284*	.495**	.284*	.444**	.476**	.711***	.523**	.711***	.47**	.711***	.422**	.711***
15	.188*	.484**	.558**	.421**	.188*	.487**	.597**	.579**	.577**	.435**	.426**	.73***	.531**	.469**	.531**	.73***
16	.328*	.556**	.328*	.419**	.493**	.771***	.502**	.771***	.328*	.52**	.328*	.467**	.498**	.771***	.591**	.771***
17	.178*	.583**	.522**	.83***	.178*	.482**	.429**	.83***	.178*	.587**	.46**	.83***	.178*	.412**	.59**	.83***
18	.347*	.347*	.347*	.347*	.51**	.591**	.517**	.481**	.53**	.464**	.462**	.444**	.742***	.742***	.742***	.742***
19	.168*	.168*	.168*	.168*	.168*	.168*	.168*	.168*	.689***	.689***	.689***	.689***	.689***	.689***	.689***	.689***
20	.307*	.307*	.428**	.538**	.307*	.307*	.524**	.578**	.535**	.547**	.855***	.855***	.504**	.532**	.855***	.855***

Note. * indicates the π_{ic} for non mastery group, ** indicates the π_{ic} for partial mastery group, *** indicates the π_{ic} for mastery group. The binary vector (e.g., 0010) under class name (e.g., C5) indicates the attribute profile of the latent class.

Table S7. Π of 4 Attributes, 20 Items, Mixed Discrimination Test.

Item	C1<br>0000	C2<br>1000	C3<br>0100	C4<br>1100	C5<br>0010	C6<br>1010	C7<br>0110	C8<br>1110	C9<br>0001	C10<br>1001	C11<br>0101	C12<br>1101	C13<br>0011	C14<br>1011	C15<br>0111	C16<br>1111
1	.378*	.579***	.378*	.579***	.378*	.579***	.378*	.579***	.378*	.579***	.378*	.579***	.378*	.579***	.378*	.579***
2	.349*	.349*	.81***	.81***	.349*	.349*	.81***	.81***	.349*	.349*	.81***	.81***	.349	.349	.81***	.81***
3	.331*	.331*	.331*	.331*	.537***	.537***	.537***	.537***	.331*	.331*	.331*	.331*	.537***	.537***	.537***	.537***
4	.269*	.269*	.269*	.269*	.269*	.269*	.269*	.269*	.786***	.786***	.786***	.786***	.786***	.786***	.786***	.786***
5	.179*	.722***	.179*	.722***	.179*	.722***	.179*	.722***	.179*	.722***	.179*	.722***	.179*	.722***	.179*	.722***
6	.393*	.393*	.654***	.654***	.393*	.393*	.654***	.654***	.393*	.393*	.654***	.654***	.393*	.393*	.654***	.654***
7	.309*	.309*	.309*	.309*	.823***	.823***	.823***	.823***	.309*	.309*	.309*	.309*	.823***	.823***	.823***	.823***
8	.246*	.246*	.246*	.246*	.246*	.246*	.246*	.246	.656***	.656***	.656***	.656***	.656***	.656***	.656***	.656***
9	.322*	.322*	.552**	.443**	.464**	.446**	.429**	.483**	.483**	.474**	.43**	.428**	.447***	.493	.716***	.716***
10	.209*	.275**	.329**	.227**	.209*	.293**	.24**	.228**	.322**	.343***	.265**	.386***	.309**	.223**	.267**	.386***
11	.24*	.24*	.24*	.24*	.854***	.854***	.854***	.854***	.24*	.24*	.24*	.24*	.854***	.854***	.854***	.854***
12	.362*	.484**	.481**	.521***	.362*	.428**	.475**	.521***	.362*	.456**	.469**	.521***	.362*	.362*	.433**	.521***
13	.281*	.476**	.523**	.47**	.422**	.449**	.534**	.484**	.558**	.421**	.487**	.597**	.579**	.577**	.435**	.683***
14	.217*	.469**	.217*	.531**	.217*	.464**	.217*	.438**	.556**	.8***	.419**	.8***	.493**	.8***	.502**	.8***
15	.298*	.441**	.37**	.431**	.298*	.435**	.389**	.359**	.32**	.438**	.343	.462***	.307**	.44**	.406**	.462***
16	.347*	.51**	.347*	.591**	.517**	.742***	.481**	.742***	.347*	.53**	.347*	.464	.462**	.742***	.444**	.742***
17	.231*	.244**	.252**	.468***	.231*	.334**	.324**	.468***	.231*	.365**	.332	.468***	.231*	.341**	.309**	.468***
18	.364*	.364*	.364*	.364*	.482**	.511**	.43**	.411**	.426**	.366**	.392	.491	.544***	.544***	.544***	.544***
19	.248*	.248*	.248*	.248*	.248*	.248*	.248*	.248*	.27***	.27***	.27***	.27***	.27***	.27***	.27***	.27***
20	.193*	.193*	.449**	.546**	.193*	.193*	.569**	.5**	.478**	.449**	.793***	.793***	.422**	.478**	.793***	.793***

Note. * indicates the π_{ic} for non mastery group, ** indicates the π_{ic} for partial mastery group, *** indicates the π_{ic} for mastery group. The binary vector (e.g., 0010) under class name (e.g., C5) indicates the attribute profile of the latent class.

Table S8. Π of 4 Attributes, 30 Items, High Discrimination Test.

Item	C1<br>0000	C2<br>1000	C3<br>0100	C4<br>1100	C5<br>0010	C6<br>1010	C7<br>0110	C8<br>1110	C9<br>0001	C10<br>1001	C11<br>0101	C12<br>1101	C13<br>0011	C14<br>1011	C15<br>0111	C16<br>1111
1	.308*	.722***	.308*	.722***	.308*	.722***	.308*	.722***	.308*	.722***	.308*	.722***	.308*	.722***	.308*	.722***
2	.327*	.327*	.752***	.752***	.327*	.327*	.752***	.752***	.327*	.327*	.752***	.752***	.327*	.327*	.752***	.752***
3	.159*	.159*	.159*	.159*	.885***	.885***	.885***	.885***	.159*	.159*	.159*	.159*	.885***	.885***	.885***	.885***
4	.328*	.328*	.328*	.328*	.328*	.328*	.328*	.328*	.782***	.782***	.782***	.782***	.782***	.782***	.782***	.782***
5	.241*	.788***	.241*	.788***	.241*	.788***	.241*	.788***	.241*	.788***	.241*	.788***	.241*	.788***	.241*	.788***
6	.241*	.241*	.889***	.889***	.241*	.241*	.889***	.889***	.241*	.241*	.889***	.889***	.241*	.241*	.889***	.889***
7	.265*	.265*	.265*	.265*	.819***	.819***	.819***	.819***	.265*	.265*	.265*	.265*	.819***	.819***	.819***	.819***
8	.33*	.33*	.33*	.33*	.33*	.33*	.33*	.33*	.676***	.676***	.676***	.676***	.676***	.676***	.676***	.676***
9	.179*	.179*	.449**	.408**	.466**	.591**	.578**	.539**	.528**	.599**	.531**	.542**	.509**	.519**	.722***	.722***
10	.224*	.593**	.58**	.538**	.224*	.559**	.405**	.496**	.552**	.443**	.464**	.753***	.446**	.429**	.483**	.753***
11	.178*	.178*	.178*	.178*	.688***	.688***	.688***	.688***	.178*	.178*	.178*	.178*	.688***	.688***	.688***	.688***
12	.191*	.447**	.493**	.79***	.191*	.453**	.572**	.79***	.191*	.409**	.488**	.79***	.191*	.56**	.424**	.79***
13	.276*	.426**	.551**	.579**	.475**	.533**	.419**	.477**	.455**	.563**	.49**	.562**	.562**	.559**	.488**	.839***
14	.284*	.542**	.284*	.4**	.284*	.495**	.284*	.444**	.476**	.711***	.523**	.711***	.47**	.711***	.422**	.711***
15	.188*	.484**	.558**	.421**	.188*	.487**	.597**	.579**	.577**	.435**	.426**	.73***	.531**	.469**	.531**	.73***
16	.328*	.556**	.328*	.419**	.493**	.771***	.502**	.771***	.328*	.52**	.328*	.467**	.498**	.771***	.591**	.771***
17	.178*	.583**	.522**	.83***	.178*	.482**	.429**	.83***	.178*	.587**	.46**	.83***	.178*	.412**	.59**	.83***
18	.347*	.347*	.347*	.347*	.51**	.591**	.517**	.481**	.53**	.464**	.462**	.444**	.742***	.742***	.742***	.742***
19	.168*	.168*	.168*	.168*	.168*	.168*	.168*	.168*	.689***	.689***	.689***	.689***	.689***	.689***	.689***	.689***
20	.307*	.307*	.428**	.538**	.307*	.307*	.524**	.578**	.535**	.547**	.855***	.855***	.504**	.532**	.855***	.855***
21	.238*	.895***	.238*	.895***	.238*	.895***	.238*	.895***	.238*	.895***	.238*	.895***	.238*	.895***	.238*	.895***
22	.199*	.462**	.199*	.482**	.402**	.437**	.569**	.446**	.448**	.415**	.449**	.546**	.569**	.747***	.5**	.747***
23	.194*	.422**	.194*	.478**	.514**	.443**	.489**	.444**	.5**	.471**	.53**	.475**	.471**	.835***	.507**	.835***
24	.256*	.256*	.483**	.453**	.256*	.256*	.526**	.437**	.573**	.549**	.743***	.743***	.534**	.524**	.743***	.743***
25	.266*	.266*	.266*	.266*	.869***	.869***	.869***	.869***	.266*	.266*	.266*	.266*	.869***	.869***	.869***	.869***
26	.243*	.568**	.462**	.542**	.453**	.519**	.496**	.453**	.513**	.583**	.58**	.455**	.464**	.597**	.524**	.884***
27	.282*	.282*	.282*	.282*	.282*	.282*	.282*	.282*	.752***	.752***	.752***	.752***	.752***	.752***	.752***	.752***
28	.201*	.201*	.43**	.515**	.448**	.592**	.52**	.503**	.481**	.576**	.473**	.458**	.434**	.434**	.771***	.771***
29	.342*	.342*	.443**	.535**	.342*	.342*	.41**	.54**	.47**	.482**	.721***	.721***	.564**	.584**	.721***	.721***
30	.236*	.546**	.236*	.537**	.236*	.411**	.236*	.479**	.496**	.805***	.512**	.805***	.54**	.805***	.583**	.805***

Note. * indicates the π_{ic} for non mastery group, ** indicates the π_{ic} for partial mastery group, *** indicates the π_{ic} for mastery group. The binary vector (e.g., 0010) under class name (e.g., C5) indicates the attribute profile of the latent class.

Table S9. Π of 4 Attributes, 30 Items, Mixed Discrimination Test.

Item	C1<br>0000	C2<br>1000	C3<br>0100	C4<br>1100	C5<br>0010	C6<br>1010	C7<br>0110	C8<br>1110	C9<br>0001	C10<br>1001	C11<br>0101	C12<br>1101	C13<br>0011	C14<br>1011	C15<br>0111	C16<br>1111
1	.393*	.654***	.393*	.654***	.393*	.654***	.393*	.654***	.393*	.654***	.393*	.654***	.393*	.654***	.393*	.654***
2	.309*	.309*	.823***	.823***	.309*	.309*	.823***	.823***	.309*	.309*	.823***	.823***	.309*	.309*	.823***	.823***
3	.205*	.205*	.205*	.205*	.343***	.343***	.343***	.343***	.205*	.205*	.205*	.205*	.343***	.343***	.343***	.343***
4	.193*	.193*	.193*	.193*	.193*	.193*	.193*	.193*	.84***	.84***	.84***	.84***	.84***	.84***	.84***	.84***
5	.196*	.73***	.196*	.73***	.196*	.73***	.196*	.73***	.196*	.73***	.196*	.73***	.196*	.73***	.196*	.73***
6	.229*	.229*	.349***	.349***	.229*	.229*	.349***	.349***	.229*	.229*	.349***	.349***	.229*	.229*	.349***	.349***
7	.224*	.224*	.224*	.224*	.753***	.753***	.753***	.753***	.224*	.224*	.224*	.224*	.753***	.753***	.753***	.753***
8	.178*	.178*	.178*	.178*	.178*	.178*	.178*	.178*	.688***	.688***	.688***	.688***	.688***	.688***	.688***	.688***
9	.225*	.225*	.447**	.493**	.453**	.572**	.409**	.488**	.56**	.424**	.512**	.441**	.426**	.551**	.874***	.874***
10	.333*	.347**	.391**	.374**	.333*	.455***	.4**	.445**	.455***	.452**	.399**	.455***	.446**	.427**	.44**	.455***
11	.194*	.194*	.194*	.194*	.769***	.769***	.769***	.769***	.194*	.194*	.194*	.194*	.769***	.769***	.769***	.769***
12	.276*	.368**	.329**	.469***	.276*	.293**	.313**	.469***	.276*	.376**	.339**	.469***	.276*	.394**	.291**	.469***
13	.217*	.597**	.579**	.577**	.435**	.426**	.531**	.469**	.531**	.464**	.438**	.556**	.419**	.493**	.502**	.8***
14	.21*	.498**	.21*	.591**	.21*	.497**	.21*	.578**	.583**	.884***	.522**	.884***	.482**	.884***	.429**	.884***
15	.212*	.354**	.32**	.233**	.212*	.295**	.355**	.3**	.273**	.309**	.26**	.5***	.258**	.245**	.268**	.5***
16	.282*	.431**	.282*	.418**	.428**	.78***	.538**	.78***	.282*	.524**	.282*	.578**	.535**	.78***	.547**	.78***
17	.364*	.482**	.511**	.544***	.364*	.43**	.411**	.544***	.364*	.426**	.366**	.544***	.364*	.392**	.491**	.544***
18	.248*	.248*	.248*	.248*	.259**	.285**	.358**	.375**	.322**	.306**	.285**	.264**	.438***	.438***	.438***	.438***
19	.314*	.314*	.314*	.314*	.314*	.314*	.314*	.314*	.377***	.377***	.377***	.377***	.377***	.377***	.377***	.377***
20	.194*	.194*	.489**	.444**	.194*	.194*	.5**	.471**	.53**	.475**	.835***	.835***	.471**	.507**	.835***	.835***
21	.203*	.753***	.203*	.753***	.203*	.753***	.203*	.753***	.203*	.753***	.203*	.753***	.203*	.753***	.203*	.753***
22	.203*	.526**	.203*	.437**	.573**	.549**	.534**	.524**	.474**	.506**	.575**	.516**	.568**	.827***	.462**	.827***
23	.282*	.519**	.282*	.496**	.453**	.513**	.583**	.58**	.455**	.464**	.597**	.524**	.587**	.752***	.493**	.752***
24	.208*	.208*	.43**	.515**	.208*	.208*	.448**	.592**	.52**	.503**	.741***	.741***	.481**	.576**	.741***	.741***
25	.184*	.184*	.184*	.184*	.693***	.693***	.693***	.693***	.184*	.184*	.184*	.184*	.693***	.693***	.693***	.693***
26	.229*	.496**	.451**	.443**	.535**	.41**	.54**	.47**	.482**	.564**	.584**	.457**	.592**	.546**	.537**	.663***
27	.262*	.262*	.262*	.262*	.262*	.262*	.262*	.262*	.769***	.769***	.769***	.769***	.769***	.769***	.769***	.769***
28	.282*	.282*	.54**	.583**	.524**	.486**	.508**	.412**	.452**	.479**	.44**	.566**	.431**	.561**	.787***	.787***
29	.234*	.234*	.329**	.281**	.234*	.234*	.343**	.294**	.38**	.379**	.412***	.412***	.343**	.273**	.412***	.412***
30	.319*	.359**	.319*	.398**	.319*	.436**	.319*	.344**	.379**	.591***	.389**	.591***	.449**	.591***	.457**	.591***

Note. * indicates the π_{ic} for non mastery group, ** indicates the π_{ic} for partial mastery group, *** indicates the π_{ic} for mastery group. The binary vector (e.g., 0010) under class name (e.g., C5) indicates the attribute profile of the latent class.
